# Can central venous pressure help identify acute right ventricular dysfunction in mechanically ventilated critically ill patients?

**DOI:** 10.1186/s13613-024-01352-9

**Published:** 2024-07-20

**Authors:** Hongmin Zhang, Hui Lian, Qing Zhang, Hua Zhao, Xiaoting Wang

**Affiliations:** 1 Department of Health Care, Peking Union Medical College Hospital, Chinese Academy of Medical Sciences and Peking Union Medical College, 1# Shuai Fu Yuan, Dong Cheng District, Beijing, 100730 China; 2 Department of Critical Care Medicine, Peking Union Medical College Hospital, Chinese Academy of Medical Sciences and Peking Union Medical College, 1# Shuai Fu Yuan, Dong Cheng District, Beijing, 100730 China

**Keywords:** Central venous pressure, Echocardiography, Right ventricular dysfunction, Critically ill

## Abstract

**Objective:**

To investigate the relationship between central venous pressure (CVP) and acute right ventricular (RV) dysfunction in critically ill patients on mechanical ventilation.

**Methods:**

This retrospective study enrolled mechanically ventilated critically ill who underwent transthoracic echocardiographic examination and CVP monitoring. Echocardiographic indices including tricuspid annular plane systolic excursion (TAPSE), fractional area change (FAC), and tricuspid lateral annular systolic velocity wave (S’) were collected to assess RV function. Patients were then classified into three groups based on their RV function and presence of systemic venous congestion as assessed by inferior vena cava diameter (IVCD) and hepatic vein (HV) Doppler: normal RV function (TAPSE ≥ 17 mm, FAC ≥ 35% and S’ ≥9.5 cm/sec), isolated RV dysfunction (TAPSE < 17 mm or FAC < 35% or S’ <9.5 cm/sec with IVCD ≤ 20 mm or HV S ≥ D), and RV dysfunction with congestion (TAPSE < 17 mm or FAC < 35% or S’ <9.5 cm/sec with IVCD > 20 mm and HV S < D).

**Results:**

A total of 518 patients were enrolled in the study, of whom 301 were categorized in normal RV function group, 164 in isolated RV dysfunction group and 53 in RV dysfunction with congestion group. Receiver operating characteristic analysis revealed a good discriminative ability of CVP for identifying patients with RV dysfunction and congestion(AUC 0.839; 95% CI: 0.795–0.883; *p* < 0.001). The optimal CVP cutoff was 10 mm Hg, with sensitivity of 79.2%, specificity of 69.4%, negative predictive value of 96.7%, and positive predictive value of 22.8%. A large gray zone existed between 9 mm Hg and 12 mm Hg, encompassing 95 patients (18.3%). For identifying all patients with RV dysfunction, CVP demonstrated a lower discriminative ability (AUC 0.616; 95% CI: 0.567–0.665; *p* < 0.001). Additionally, the gray zone was even larger, ranging from 5 mm Hg to 12 mm Hg, and included 349 patients (67.4%).

**Conclusions:**

CVP may be a helpful indicator of acute RV dysfunction patients with systemic venous congestion in mechanically ventilated critically ill, but its accuracy is limited. A CVP less than10 mm Hg can almost rule out RV dysfunction with congestion. In contrast, CVP should not be used to identify general RV dysfunction.

**Supplementary Information:**

The online version contains supplementary material available at 10.1186/s13613-024-01352-9.

## Background

The right ventricle (RV) has emerged as a critical determinant of haemodynamics and prognosis in recent years [1–4]. Its primary function is to deliver all the blood it receives to the pulmonary circulation on a beat-to-beat basis without causing the central venous pressure (CVP) to rise [5]. CVP may sometimes serve as a marker for the balance between venous return and the RV’s capacity for accommodation [6]. However, as an intraluminal pressure, CVP can be influenced by intrathoracic pressure, pericardial pressure and intraabdominal pressure, which are particularly more easily elevated in critically ill patients [7].

Despite the established role of RV function in haemodynamics, a knowledge gap persists regarding the optimal CVP threshold for assessing RV dysfunction [3]. Bech-Hanssen et al. suggested CVP ≥ 10 mmHg as a criterion for severe RV dysfunction in patients with left ventricular disease [8]. For patients with a left ventricular assist device, CVP over 15 or 16mmHg was considered indicative of RV failure [9, 10]. Nevertheless, Vieillard-Baron suggested that RV dilation and CVP ≥ 8 mmHg defined RV dysfunction with potential implications of volume management in patients with septic shock [11]. The limited investigation of CVP’s utility in identifying acute RV dysfunction in critically ill patients motivates this study. The aim of this study is to delineate the relationship between CVP and acute right ventricular dysfunction in critically ill patients on mechanical ventilation.

## Patients and methods

### Study population

This retrospective cohort study investigated patients admitted to the intensive care unit (ICU) of a tertiary hospital between August 2018 and January 2024. Inclusion criteria comprised mechanically ventilated critically ill patients who underwent transthoracic echocardiography (TTE) examination within the the first 24 h of admission.

We exclude patients with pre-existing chronic heart failure, cardiac surgery, moderate to severe chronic pulmonary hypertension, constrictive pericarditis, atrial fibrillation, or those lacking CVP monitoring or with inadequate TTE views.

The study was conducted in compliance with the Declaration of Helsinki and was approved by the ethics committee of our hospital (Approval No. I-23PJ1278). Written consent on the review and research of the patients’ medical data was obtained from the next of kin.

### Echocardiography

TTE was routinely performed in this critical care setting. Haemodynamic and respiratory parameters were recorded concurrently during the examination. Images were archived for offline analysis. A Mindray echocardiograph (Shenzhen, China) equipped with a 2.5-MHz phased-array probe was used for image acquisition. The echocardiographic findings were interpreted according to the PRICES statement [12]. Two physicians with more than 10 years of echocardiograhic experience independently reviewed the images and reached consensus on the results. Intraobserver and interobserver variability for key cardiac function measurements by these investigators have been previously reported [13].

RV function was assessed using tricuspid annular plane systolic excursion (TAPSE), fractional area change (FAC) and tricuspid lateral annular systolic velocity wave (S’). All measurements were obtained from the apical 4-chamber view. TAPSE was measured by placing the M-mode cursor along the lateral part of the tricuspid valve ring. S’ was measured by placing the sample volume on the lateral part of the tricuspid valve ring with pulsed-wave tissue Doppler. FAC was calculated as [(end-diastolic area―end-systolic area)/end-diastolic area]×100. RV dysfunction was defined as TAPSE < 17 mm or FAC < 35% or S’ <9.5 cm/Sec. [14]. R/LVEDA ratio > 0.6 was considered RV dilation [4]. Acute cor pulmonale (ACP) was defined as RV dilation in combination with septal paradoxical motion at end-systole [15].

Systemic venous congestion was evaluated using inferior vena cava diameter (IVCD) and the hepatic vein (HV) Doppler waveforms. An IVCD > 20 mm and an HV spectral Doppler pattern demonstrating an S wave velocity lower than the D wave velocity (S < D) were indicative of congestion [16]. The IVCD was measured in the subcostal longitudinal view at the end of expiration, just upstream of the origin of the hepatic vein. The HV was also identified from the subcostal view by positioning a sample volume at 2–3 cm from its junction with the IVC.

Left ventricular outflow tract velocity-time integral (LVOT-VTI), left ventricular ejection fraction (LVEF), mitral peak E velocity (E), averaged tissue Doppler velocity of lateral and medial mitral annuli at early diastole (e’), tricuspid regurgitation (TR), stroke volume index and cardiac index were obtained using previously described methods [13].

Patients were subsequently divided into three clinically relevant groups based on RV function and systemic venous congestion: (1) Normal RV group (TAPSE ≥ 17 mm, FAC ≥ 35% and S’ ≥9.5 cm/sec); (2) Isolated RV dysfunction group (TAPSE < 17 mm or FAC < 35% or S’ <9.5 cm/sec, with IVCD ≤ 20 mm or HV S ≥ D); and (3) RV dysfunction + congestion group (TAPSE < 17 mm or FAC < 35% or S’ <9.5 cm/sec, with IVCD > 20 mm and HV S < D).

### Clinical data collected

We collected the patients’ demographic information, baseline Acute Physiology and Chronic Health Evaluation (APACHE) II score, and Sequential Organ Failure Assessment (SOFA) score. Additionally, heart rate (HR), mean arterial pressure (MAP), norepinephrine (NE) dose, PEEP, plateau pressure (Pplat) and fluid balance at the time of echocardiogram were obtained either from the data record during echocardiography or from the medical record. CVP was measured at the end-expiratory phase with the patient in the supine position and the transducer zeroed at the mid-thoracic level exactly after the echocardiographic examination. We also collected data on 30-day mortality, maximum lactate level within the first 24 h and the incidence of acute respiratory distress syndrome (ARDS) and acute kidney injury (AKI) within the first 24 h. AKI was defined as an increase in serum creatinine of at least 26 µmol/L increase or a 50% increase, or the initiation of renal replacement therapy Using the Kidney Disease Improving Global Outcomes (KDIGO) consensus criteria [17]. We excluded urine output criteria due to potential confounding by diuretic use, which is common in ICU patients.

### Statistical analysis

Baseline demographics and echocardiographic measurements were reported as median (interquartile range) for quantitative variables and number (percentage) for qualitative variables or as the mean ± SD. The distributions of the continuous values were assessed for normality using the Kolmogorov-Smirnov test. The Kruskal‒Wallis test was used to compare quantitative variables, with a Dunnett’s post-hoc test for pairwise comparisons if necessary. Categorical variables were compared using the chi-squared test, or Fisher’s exact test, as appropriate. Spearman’s rank correlation coefficient assessed correlations. Receiver operating characteristic (ROC) curves with 95% confidence interval (CI) were generated to evaluate CVP’s ability to detect both RV dysfunction with congestion and general RV dysfunction. The Youden index was used to identify the optimal cutoff value. Gray zones were calculated using two methods: (1) the 95% CI of the Youden’s index from a 1000 population bootstrap, and (2) cut-off values corresponding to a sensitivity or specificity of less than 90% (10% diagnosis tolerance) [18]. The largest interval from these two methods defined the gray zone. Binary logistic regression identified predictors of RV dysfunction with congestion. Baseline covariates with *p* values < 0.1 from the univariate model were included in the multivariate model and odds ratio with 95% CIs were calculated. All statistical analyses were performed using SPSS (SPSSInc., Chicago, Ill., USA) and Graphpad Prism (version 6.01 for Windows, GraphPad Software, La Jolla California USA). A two-tailed *p* < 0.05 was considered significant.

## Results

### General characteristics

**Fig. 1:**
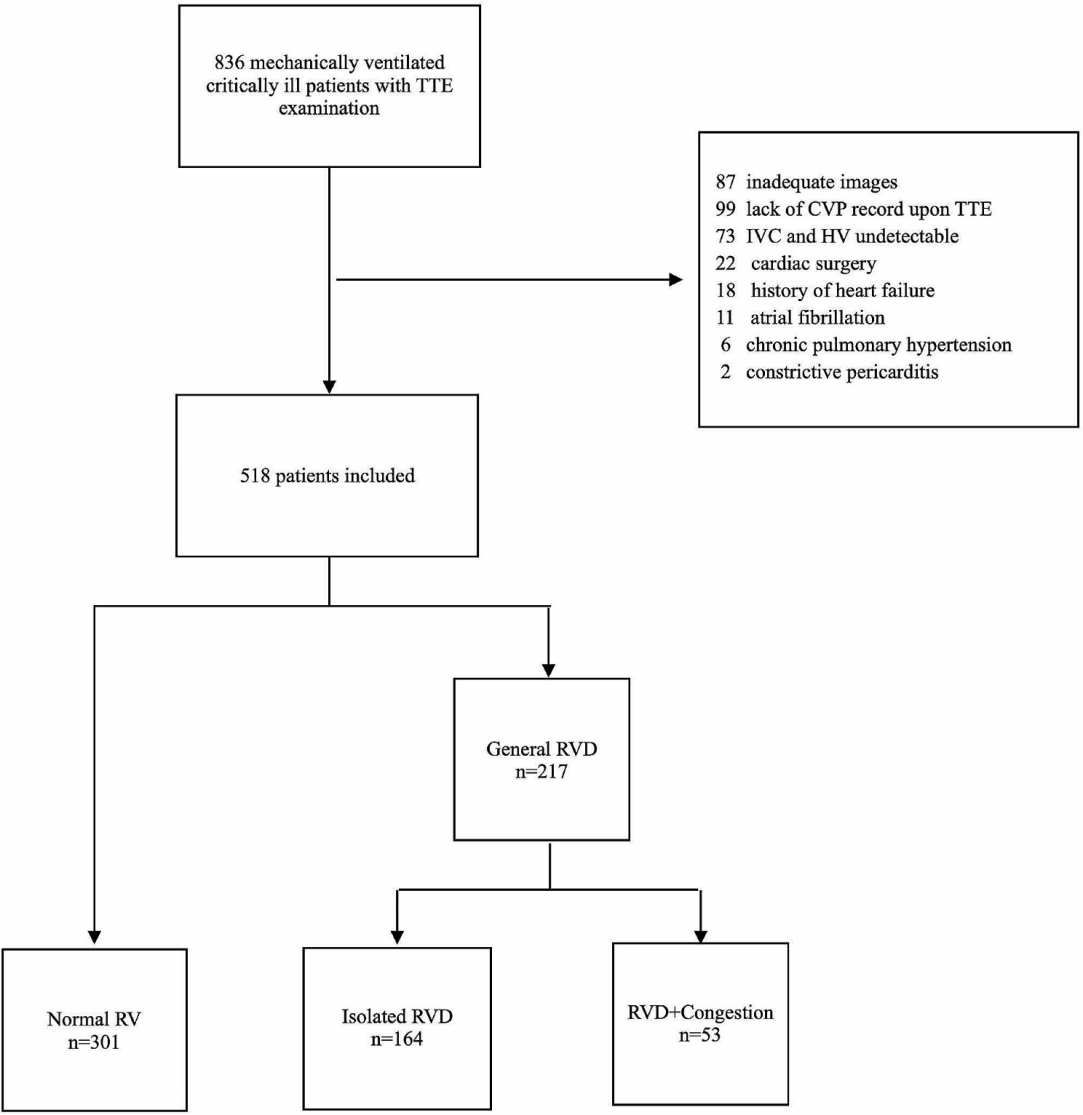
Flow chart of this study. TTE: transthoracic echocardiography; CVP: central venous pressure; IVC: inferior vena cava; HV: hepatic vein; RV: right ventricle; RVD: right ventricular dysfunction

**Fig. 2:**
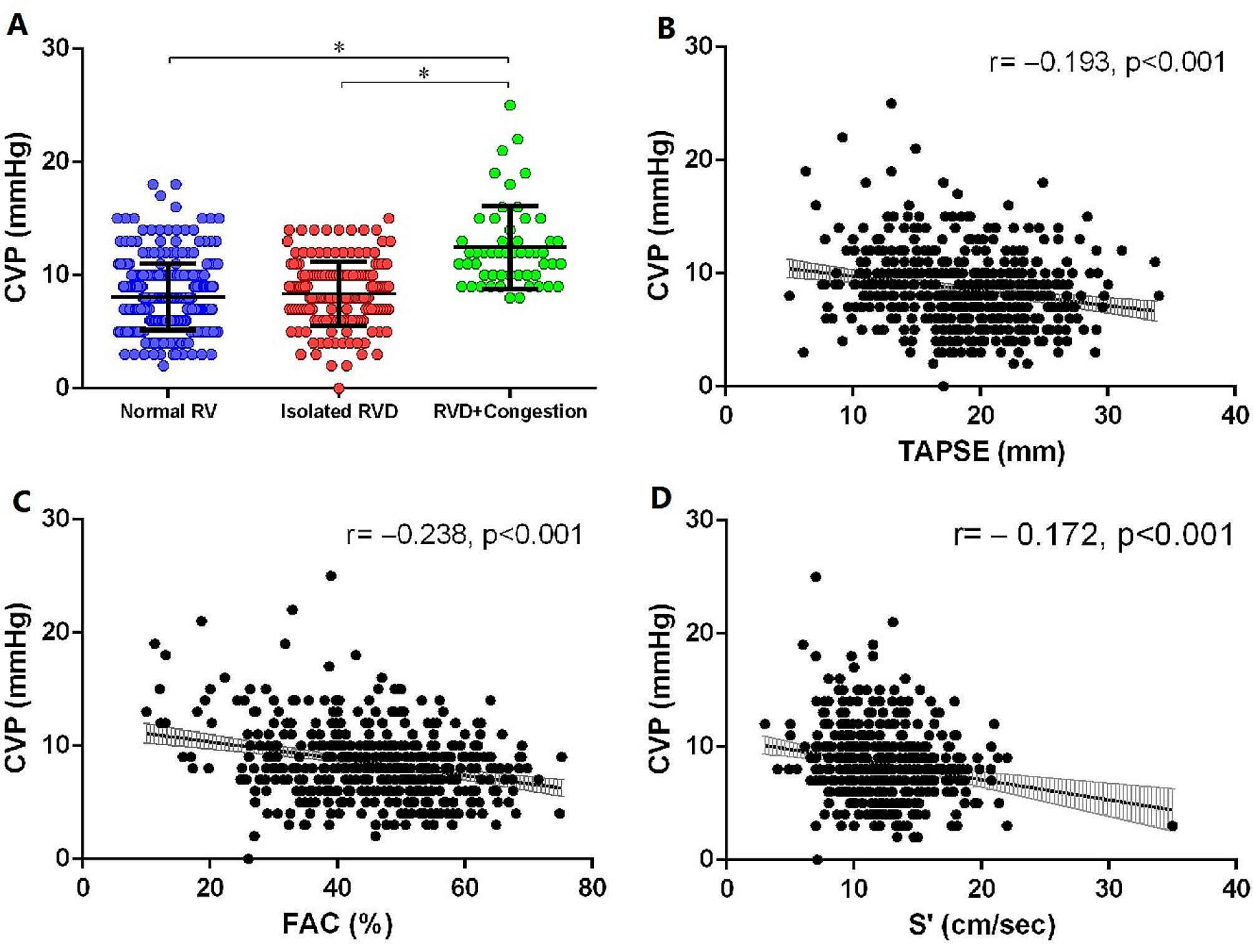
**A**. Relationship between CVP and RV function. The RVD + Congestion group had significantly greater CVP than isolated RVD group and normal RV function group, *p* < 0.001. **B**. TAPSE correlated with CVP, *r*=-0.193, *p* < 0.001. **C**. FAC correlated with CVP, *r*=-0.238, *p* < 0.001. **D**. RV S’ correlated with CVP, *r*=-0.172, *p* < 0.001CVP: central venous pressure; RV: right ventricle; RVD: right ventricular dysfunction; TAPSE: tricuspid annular plane systolic excursion; FAC: fractional area change; S’: tricuspid lateral annular systolic velocity wave

A total of 836 patients were assessed for eligibility, of which 318 were excluded (Fig. 1). Among the 518 enrolled patients, 301 had normal RV function, 164 had isolated RV dysfunction, and 53 had RV dysfunction with congestion. The three groups differed significantly in baseline characteristics including APACHE II, SOFA, NE dose, HR, Pplat level, maximum lactate level in 24 h, fluid balance, ICU-free days, ARDS occurrence, AKI occurrence, and 30-day mortality (all *p* < 0.05) (Table 1). The three groups had significantly different levels of CVP (*p* < 0.001) (Fig. 2A).

**Table 1:** General characteristics of all the patients

Categories	All patients (*n* = 518)	Normal RV (*n* = 301)	Isolated RVD (*n* = 164)	RVD + Congestion (*n* = 53)	*p* value
Age, years	63 (51, 73)	62 (50, 72)	65 (54, 77)	66 (57, 75)	0.062
Male gender (n, %)	317 (61.2)	174 (57.8)	104 (63.4)	39 (73.6)	0.073
APACHEII	21 (16, 27)	20 (15, 27)	22 (17, 28)	25 (19, 30)	0.009
SOFA	11 (9, 14)	11 (9, 13)	13 (10, 14)	12 (11, 15)	<0.001
Septic shock (n, %)	211 (40.7)	117 (38.9)	70 (42.7)	24 (45.2)	0.564
Nonseptic shock (n, %)	22 (4.2)	9 (3.0)	9 (5.5)	4 (7.5)	0.201
ARDS (n, %)	74 (14.3)	24 (8.0)	25 (15.2)	25 (47.2)	<0.001
AKI (n, %)	253 (48.8)	122 (40.5)	94 (57.3)	37 (69.8)	<0.001
Comorbidities (n, %)					
HTN	185 (35.7)	98 (32.6)	64 (39.0)	23 (43.3)	0.178
DM	80 (15.4)	44 (14.6)	30 (18.3)	6 (11.3)	0.393
CAD	69 (13.3)	39 (13.0)	24 (14.6)	6 (11.3)	0.793
NE infusion (n, %)	407 (78.6)	221 (73.4)	139 (84.8)	47 (88.7)	0.003
NE dose (µg/kg/min)	0.3 (0.1, 0.5)	0.2 (0.1, 0.5)	0.3 (0.2, 0.7)	0.5 (0.2, 0.9)	<0.001
HR (bpm)	92 ± 19	90 ± 19	95 ± 20	100 ± 20	0.003
MAP (mm Hg)	79 (72, 86)	80 (74, 88)	81(71, 85)	75(67, 83)	0.009
CVP (mm Hg)	8 (6, 10)	8 (6, 10)	8 (7, 10)	12 (10, 13)	<0.001
PEEP (cmH_2_O)	6 (5, 8)	6 (5, 8)	6 (5, 8)	7(5, 8)	0.449
Pplat (cmH_2_O)	18 (16, 21)	18 (16, 21)	19 (16, 21)	20 (18, 22)	0.006
PaO_2_/FiO_2_ (mm Hg)	253 (198, 319)	264 (212, 341)	252 (175, 302)	204 (126, 262)	<0.001
PaCO_2_ (mm Hg)	39 (36, 42)	39 (35, 42)	39 (36, 43)	40 (37, 43)	0.481
*Max lactate (mmol/L)	2.5 (1.5, 4.4)	2.4 (1.5, 4.1)	3.0 (1.9, 5.3)	3.0 (1.6, 7.2)	0.025
Fluid balance (ml)	-162 (-1099, 732)	-123 (-1220, 549)	260 (-727, 826)	-403 (-2353, 1299)	0.142
ICU-free days	22 (7, 26)	23 (11, 26)	19 (0, 25)	7 (0, 20)	<0.001
30-day mortality (n, %)	95 (18.3)	37 (12.3)	34 (20.7)	24 (45.3)	<0.001

*maximum lactate within the first 24 h

APACHE: acute physiology and chronic health evaluation; SOFA: sequential organ failure assessment; ARDS: acute respiratory distress syndrome; HTN: hypertension; DM: diabetes mellitus; CAD: coronary artery disease; NE: norepinephrine; HR: heart rate; MAP: mean arterial pressure; CVP: central venous pressure; PEEP: positive end-expiratory pressure; Pplat: plateau pressure; AKI: acute kidney injury; ICU: intensive care unit

### Comparison of echocardiographic parameters among the three groups

The three groups exhibited significant differences in LVEF, MAPSE, E velocity, TAPSE, FAC, RV S’, lateral e’, medial e’, LVOT-VTI, and IVCD (*p* < 0.001). Additionally, significant differences were observed in R/LVEDA, TR, E/e’ and cardiac index (*p* < 0.05). The group with RVD and congestion had the highest prevalence of ACP (*p* < 0.001) (Table 2).

**Table 2:** Echocardiographic parameters

Categories	Number of missing values	All patients (*n* = 518)	Normal RV (*n* = 301)	Isolated RVD (*n* = 164)	RVD + Congestion (*n* = 53)	*p* value
LVEF(%)	4	58 (47, 66)	61 (53, 69)	49 (40, 60)	47(36, 61)	<0.001
E(cm/sec)	29	66 (54, 81)	65 (54, 78)	59 (47, 76)	70 (58, 95)	<0.001
Lateral e’(cm/sec)	42	8.7 (6.6, 9.9)	9.3 (7.2, 11.7)	7.8 (5.9, 10.0)	8.1 (6.0, 10.1)	<0.001
Medial e’(cm/sec)	52	6.4 (5.0, 8.1)	6.9 (5.6, 8.8)	5.4 (4.3, 6.9)	6.3 (4.7, 8.5)	<0.001
Average E/e’	52	8.6 (6.7, 11.1)	8.2 (6.7, 10.2)	9.6 (6.6, 10.8)	9.9 (7.5, 13.5)	0.002
TAPSE (mm)	1	19.2 ± 5.0	21.9 ± 3.3	14.8 ± 3.3	13.0 ± 3.5	<0.001
RV FAC (%)	60	47 (38, 54)	51 (44, 57)	36 (30, 47)	32 (26, 42)	<0.001
RV S’ (cm/sec)	29	12.0 (9.9, 14.3)	13.2 (11.7, 15.3)	9.6 (8.4, 11.6)	9.1 (7.2, 11.2)	<0.001
TR (m/sec)	69	2.3 (2.1, 2.6)	2.3 (2.1, 2.5)	2.3 (2.0, 2.6)	2.5 (2.3, 2.8)	0.001
R/LVEDA	41	0.52 (0.45, 0.62)	0.52 (0.45, 0.60)	0.51 (0.45, 0.62)	0.61 (0.49, 0.68)	0.017
ACP (n, %)	0	19 (3.7)	2 (0.1)	6 (3.7)	11 (20.8)	<0.001
LVOT-VTI(cm)	28	16.5 (13.6, 19.3)	17.2 (15.0, 19.9)	14.4 (11.8, 16.8)	12.7 (11.4, 14.9)	<0.001
SVI (ml/min/m^2^)	30	34.0 (26.9, 40.5)	35.5 (29.1, 41.6)	29.3 (23.3, 35.6)	26.7 (22.5, 33.6)	<0.001
CI (L/min/m^2^)	30	3.0 (2.5, 3.6)	3.0 (2.6, 3.8)	2.7 (2.1, 3.3)	2.7 (2.1, 3.4)	0.012
IVCD (mm)	0	17.9 (14.3, 21.0)	17.1 (14.0, 20.2)	17.6 (15.0, 20.3)	21.9 (20.3, 23.9)	<0.001
HV S < D (n, %)	0	79 (15.3)	13 (4.3)	13 (7.9)	53(100)	<0.001

LVEF: left ventricular ejection fraction; E: mitral peak E velocity; TAPSE: tricuspid annular plane systolic excursion; FAC: fractional area change; S’: tricuspid lateral annular systolic velocity wave; TR: tricuspid regurgitation; R/LVEDA: right and left end-diastolic area ratio; ACP: acute cor pulmonale; LVOT-VTI: left ventricular outflow tract velocity-time integral; SVI: stroke volume index; CI: cardiac index; IVCD: diameter of inferior vena cava; HV: hepatic vein

### Correlation analysis of CVP and RV function

We observed weak correlations between CVP and TAPSE, RV FAC and RV S’ (*r*=-0.193,-0.238,-0.172, respectively; all *p* < 0.001) (Fig. 2B and D).

### ROC analysis of CVP for the detection of RV dysfunction

**Fig. 3:**
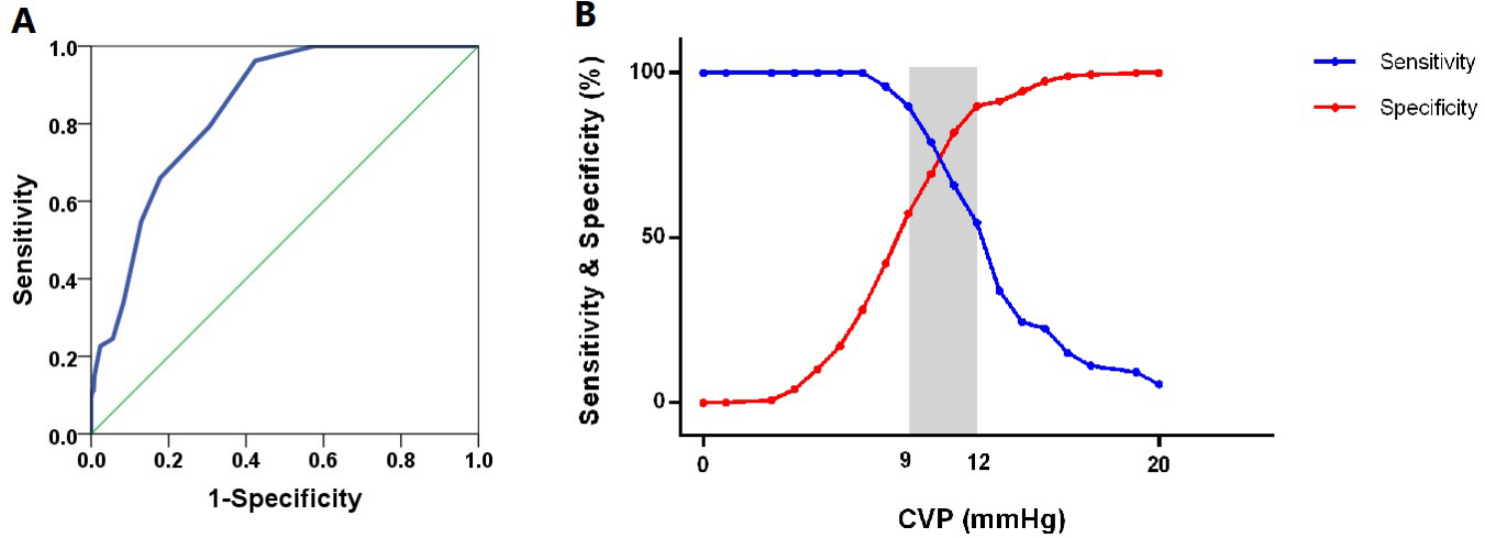
ROC curve and sensitivity and specificity of CVP to detect RVD + Congestion. **A**. ROC curve for the CVP to detect RVD + Congestion. The AUC of CVP for determining RV dysfunction + systemic venous congestion was 0.839 in all patients, 95% CI: 0.795–0.883; *p* < 0.001. **B**. Sensitivity and specificity of CVP to detect RVD + Congestion. The inconclusive zone, which is > 10% diagnosis tolerance, is represented as a shaded rectangle. The gray zone ranged between 9 mmHg and 12 mmHg, in which 95 (18.3%) patients were situated CVP: central venous pressure; RVD: right ventricular dysfunction;

The ROC analysis showed that the AUC of CVP for determining RV dysfunction with congestion was 0.839 in all patients (95% CI: 0.795–0.883; *p* < 0.001). A cutoff of 10 mm Hg yielded a sensitivity of 79.2%, and a high negative predictive value (NPV) of 96.7%, indicating its utility in ruling out this combined condition. However, the positive predictive value (PPV) was 22.8%. The gray zone ranged between 9 mm Hg and 12 mm Hg, in which 95 patients (18.3%) were situated (Table 3; Fig. 3A and B).

**Table 3:** ROC analysis of CVP in the prediction of RVD + congestion and general RVD

	AUC	95%CI	*p*	Optimal cutoff (mm Hg)	Sensitivity	Specificity	PPV	NPV
RVD + congestion	0.839	0.795–0.883	< 0.001	10	79.2%	69.4%	22.8%	96.7%
General RVD	0.616	0.567–0.665	< 0.001	9	58.5%	59.8%	51.3%	66.6%

CVP: central venous pressure; RVD: right ventricular dysfunction; PPV: positive predictive value; NPV: negative predictive value

**Fig. 4:**
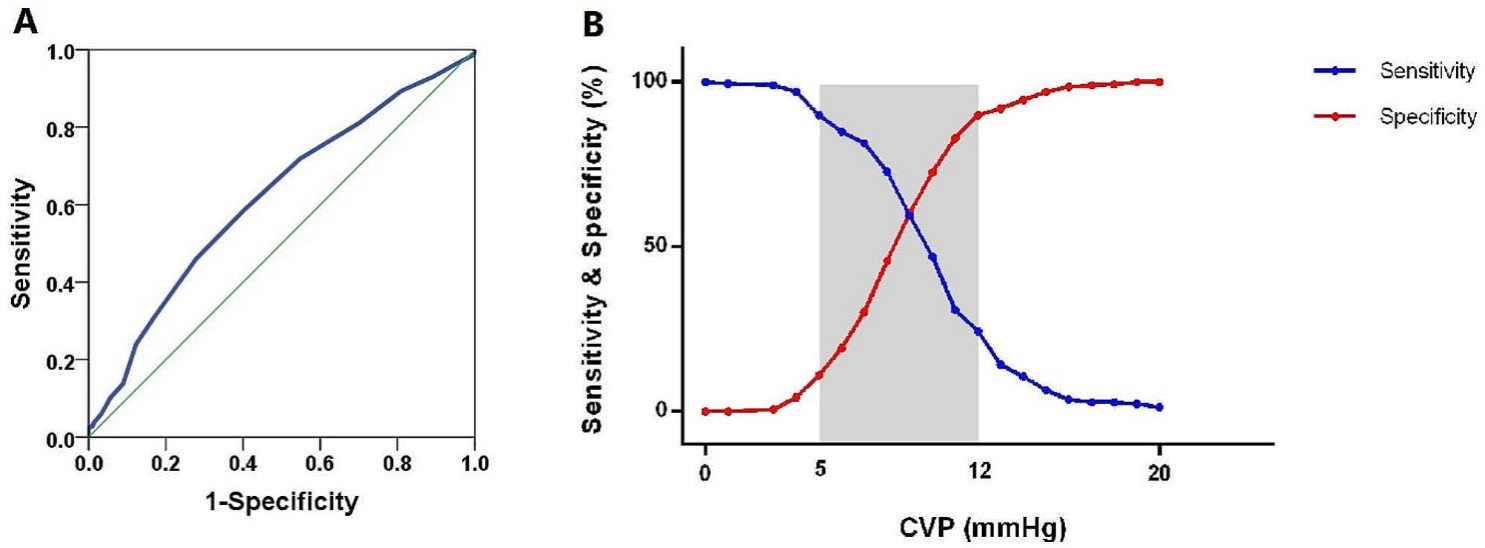
ROC curve and sensitivity and specificity of CVP to detect general RV dysfunction. **A**. ROC curve for the CVP to detect general RVD. The AUC of CVP for determining RVD was 0.616 in all patients, 95% CI: 0.567–0.665; *p* < 0.001. **B**. Sensitivity and specificity of CVP to detect general RVD. The inconclusive zone, which is > 10% diagnosis tolerance, is represented as a shaded rectangle. The gray zone ranged between 5 mmHg and 12 mmHg, in which 349 (67.4%) patients were situated. CVP: central venous pressure; RVD: right ventricular dysfunction;

CVP demonstrated a lower discriminative ability (AUC 0.616; 95% CI: 0.567–0.665; *p* < 0.001) for identifying all patients with RV dysfunction compared to RV dysfunction with congestion, as shown by ROC analysis. The optimum cutoff was 9 mm Hg, offering a balanced sensitivity (58.5%) and specificity (59.8%). However, a substantial proportion of patients (67.4%, *n* = 349) fell within the gray zone (5–12 mm Hg), further emphasizing the limitations of CVP as a single diagnostic tool for overall RV dysfunction (Table 3; Fig. 4A and B).

### Multivariate analysis

Multivariate logistic regression analysis, adjusting for the presence of ARDS, NE dose, and Pplat level, revealed that CVP (OR 1.494, 95%CI: 1.312–1.702; *p* < 0.001) and the presence of ARDS (OR 2.494, 95%CI: 1.118–5.565; *p* < 0.001) were independent predictors of RV dysfunction with congestion (Supplemental Table 1).

### Sensitivity analysis

To assess the influence of LVEF on the diagnostic ability of CVP for detecting RV dysfunction with congestion, a sensitivity analysis was performed. Among the 341 patients with LVEF ≥ 50%, 20 patients had confirmed RV dysfunction with congestion; Similarly, among the 177 patients with LVEF < 50%, 33 patients had RV dysfunction with congestion. Sensitivity analysis reviewed similar discriminative ability of CVP to detect RV dysfunction with congestion in patients with LVEF ≥ 50% compared to those with LVEF < 50% (AUC 0.864 vs. 0.788, Z = 1.590, *p* = 0.112). Likewise, CVP demonstrated similar discriminative ability in patients with R/LVEDA ≥ 0.6 compared to those with R/LVEDA < 0.6 (AUC 0.883 vs. 0.816, Z = 1.373, *p* = 0.170).

## Discussion

This study investigated the utility of CVP in identifying acute RV dysfunction with systemic venous congestion in mechanically ventilated critically ill patients. While CVP demonstrated acceptable discriminative ability for this combined endpoint (AUC = 0.839), a large gray zone (18.3%) limited its diagnostic accuracy. Furthermore, CVP was not a reliable marker for general RV dysfunction based solely on systolic function. These findings suggest that CVP may be a helpful adjunct for identifying RV dysfunction with congestion, but its limitation necessitates a cautious approach, particularly when used alone.

CVP measurement is a routine procedure in ICUs, readily available for critically ill patients. While its role in guiding fluid resuscitation has been debated, CVP can still serve as a “stopping sign” to avoid excessive fluid administration [19–21]. If the CVP is high, the pressure in the upper venous reservoir and capillary is even higher, which will markedly increase edema formation [22]. Thus, more attention has been paid on the CVP’s value of indicating volume overload and subsequent organ perfusion impairment [23, 24]. However, CVP can reflect the interplay between venous return and RV function. When RV function deteriorates, it cannot adequately handle venous return, leading to elevated CVP. Given the importance of timely RV dysfunction detection, echocardiography presents challenges due to complex parameters, image acquisition and interpretation. Therefore, a readily available tool like CVP, if indicative of RV dysfunction, could serve as an alert for clinicians.

While a standardized RV dysfunction criteria is lacking, we employed three commonly used echocardiographic indices. We found that ARDS was an independent predictor of RV dysfunction with congestion, potentially fulfilling RV failure criteria established by the European Society of Cardiology [25]. ARDS is one of the most common conditions to challenge the RV, depending on the severity of lung injury and ventilator settings [4]. While our ICU population was heterogeneous, 47.2% of patients with RV dysfunction and congestion had ARDS and 20.8% had ACP. Elevated CVP can also indicate RV dysfunction with congestion, however, the wide range around the CVP cut-off value and the low positive predictive value (22.8%) highlights its limitations as a sole diagnostic tool. This may be explained by the influence of intrathoracic pressure on CVP in critically ill patients, even though no significant differences were observed in ventilator settings among groups. A future study incorporating esophageal pressure monitoring might clarify this hypothesis. Our previous work also demonstrated elevated CVP can coexist with normal RV systolic function, as evidenced by HV Doppler S > D in some patients [26]. Notably, measurement bias cannot be entirely eliminated, further contributing to the low positive predictive value. Importantly, the high negative predictive value of 96.7% suggests that RV dysfunction with systemic congestion is very unlikely in patients with CVP below 10 mm Hg. Furthermore, a CVP upper limit value of 12 mm Hg aligns with prior studies [27–29]. These studies have shown that CVP above 13 mm Hg is unlikely to indicate fluid responsiveness and may be associated with worse outcomes in critically ill patients. Therefore, CVP should be considered a warning sign of potential RV compromise, necessitating a comprehensive evaluation that includes echocardiography. Moreover, given the prognostic significance of RV dysfunction, clinicians should strive to maintain the lowest possible CVP in critically ill patients.

We had hypothesized that CVP could reflect RV dysfunction since CVP was determined by venous return and cardiac function based on the Starling curve and Guyton theory [30]. However, our observation of similar CVP values in patients with isolated RV dysfunction and normal RV function, coupled with the substantial gray zone, underscores the limitations of CVP for detecting general RV dysfunction. This aligns with the concept that RV filling can occur below its unstressed volume without a significant change in distending pressure, and RV filling normally is independent of CVP [31]. In contrast, a rising CVP with stable or declining RV stroke volume suggests RV failure, potentially serving as a stopping point for further fluid administration. Therefore, CVP may be more indicative of RV failure rather than RV dysfunction. Furthermore, we found a higher prevalence of AKI in patients with isolated RV dysfunction compared to those with normal RV function. This could be due to patients with isolated RV dysfunction had lower CI than normal RV function patients (2.7 vs. 3.0 L/min/m^2^). Moreover, patients with isolated RV dysfunction could be more easily develop congestion as evidenced by the higher abnormal hepatic vein Doppler rates (7.9% vs. 4.3%). While the mechanism requires further investigation, this finding suggests that detection of isolated RV dysfunction may still hold clinical relevance.

A key strength of this study lies in the large size with comprehensive echocardiographic and CVP monitoring, ensuring a high measurement rate for RV-related parameters. However, this study has several limitations. First, the retrospective design limits the power of the conclusions. Additionally, the inclusion criteria restricted the study population to mechanical ventilated patients with TTE and excluded a substantial number of patients due to missing CVP data or inadequate IVC images. This may limit the generalizability of the findings. Second, while the study focused on IVC and HV measurements to assess systemic congestion, the absence of portal and intrarenal vein Doppler data may provide a less comprehensive picture of venous haemodynamics. Nonetheless, IVC and HV offer a direct connection to the right heart, reflecting RV function to a significant degree. Previous research has demonstrated a correlation between hepatic S/D ratio and portal vein pulsatility fraction in cardiac surgery patients [32]. Finally, the study design only captured CVP at a single time point. Future prospective studies with serial measurements are warranted to strengthen the conclusion. Despite these limitations, this study suggests that a CVP above 12 mm Hg in mechanically ventilated patients is associated with a high likelihood of RV failure. However, echocardiographic evaluation should be considered crucial for definitive diagnosis of RV dysfunction in patients with CVP values within the gray zone.

## Conclusions

CVP may be a helpful indicator of acute RV dysfunction patients with systemic venous congestion in mechanically ventilated critically ill, but its accuracy is limited. A CVP less than 10 mm Hg can almost rule out RV dysfunction with congestion. In contrast, CVP should not be used to identify general RV dysfunction.

### Electronic supplementary material

Below is the link to the electronic supplementary material.

Supplementary Material 1

## Data Availability

All datasets used and analyzed during the current study are available from the corresponding author on reasonable request.

## Abbreviations

RV: Right Ventricle
CVP: Central Venous Pressure
PEEP: Positive End-Expiratory Pressure
ICU: Intensive Care Unit
TTE: Transthoracic Echocardiography
TAPSE: Tricuspid Annular Plane Systolic Excursion
FAC: Fractional Area Change
R/LVEDA: Right And Left Ventricular End-Diastolic Area Ratio
IVCD: Internal Diameter Of Inferior Vena Cava
HV: Hepatic Vein
LVOT-VTI: Left Ventricular Outflow Tract Velocity-Time Integral
LVEF: Left Ventricular Ejection Fraction
E: Mitral Peak E Velocity
e’: Averaged Tissue Doppler Velocity Of Lateral And Medial Mitral Annuli At Early Diastole
TR: Tricuspid Regurgitation
APACHE: Acute Physiology And Chronic Health Evaluation
SOFA: Sequential Organ Failure Assessment
HR: Heart Rate
MAP: Mean Arterial Pressure
NE: Norepinephrine
Pplat: Plateau Pressure
AKI: Acute Kidney Injury
ROC: Receiver Operating Characteristic
CI: Confidence Interval
