# Supplementary material for: Can central venous pressure help identify acute right ventricular dysfunction in mechanically ventilated critically ill patients?
Source: Ann Intensive Care. 2024 Jul 20;14:114. doi: 10.1186/s13613-024-01352-9 (PMC11264666; doi:10.1186/s13613-024-01352-9)
Supplement: Supplementary file 1 — Supplementary Material 1 [file 13613_2024_1352_MOESM1_ESM.docx]

Supplemental Table 1. Factors associated with RV dysfunction+congestion

|  | OR | 95%CI | *p* |
| --- | --- | --- | --- |
| **Univariate analysis** |  |  |  |
| Age | 1.012 | 0.995-1.030 | 0.159 |
| Shock | 1.340 | 0.759-2.367 | 0.313 |
| ARDS | 7.562 | 4.087-13.990 | < 0.001 |
| NE | 2.044 | 1.347-3.104 | 0.001 |
| PEEP | 1.012 | 0.885-1.158 | 0.861 |
| Pplat | 1.106 | 1.032-1.184 | 0.004 |
| CVP | 1.478 | 1.331-1.641 | < 0.001 |
| **Multivariate analysis** |  |  |  |
| ARDS | 2.494 | 1.118-5.565 | 0.026 |
| CVP | 1.494 | 1.312-1.702 | < 0.001 |

RF: respiratory failure; ARDS: acute respiratory distress syndrome; NE: norepinephrine; PEEP: positive end-expiratory pressure; Pplat: plateau pressure; CVP: central venous pressure.
